# Supplementary material for: Fatty acyl-CoA reductase influences wax biosynthesis in the cotton mealybug, Phenacoccus solenopsis Tinsley
Source: Commun Biol. 2022 Oct 19;5:1108. doi: 10.1038/s42003-022-03956-y (PMC9582030; doi:10.1038/s42003-022-03956-y)
Supplement: Supplementary file 2 — Supplementary Information [file 42003_2022_3956_MOESM2_ESM.docx]

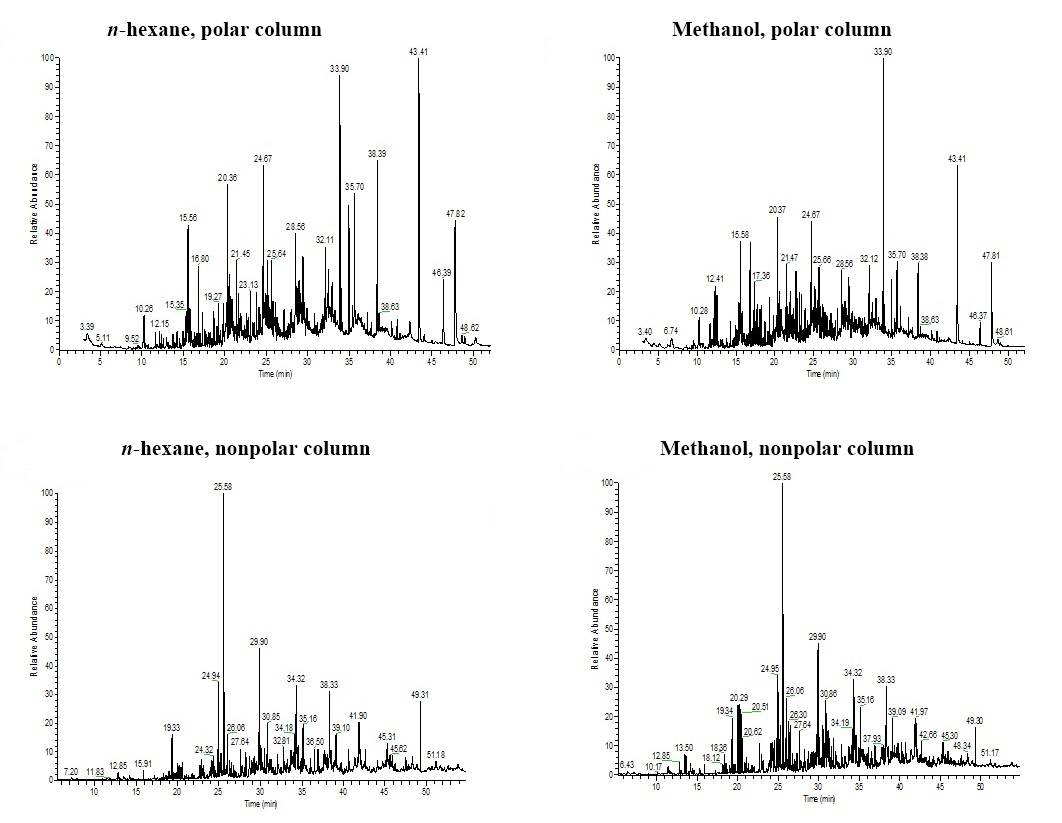
Supplementary Figure 1 GC-MS analysis of cotton mealybug wax dissolved in *n*-hexane and methanol and tested on polar and nonpolar chromatographic columns. The solvent and column type are showed on the top of each peak figure.


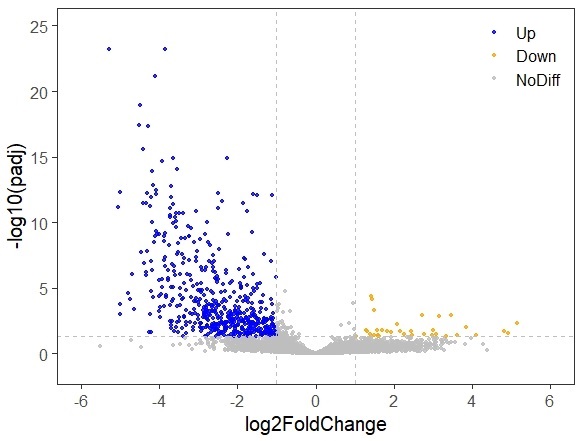


Supplementary Figure 2 Volcano plot showing DEGs between the integument and other tissues. Blue circles show 527 upregulated DEGs in the integument, yellow circles show 34 downregulated DEGs in the integument, and genes that are not significantly differentially expressed are represented by grey circles.


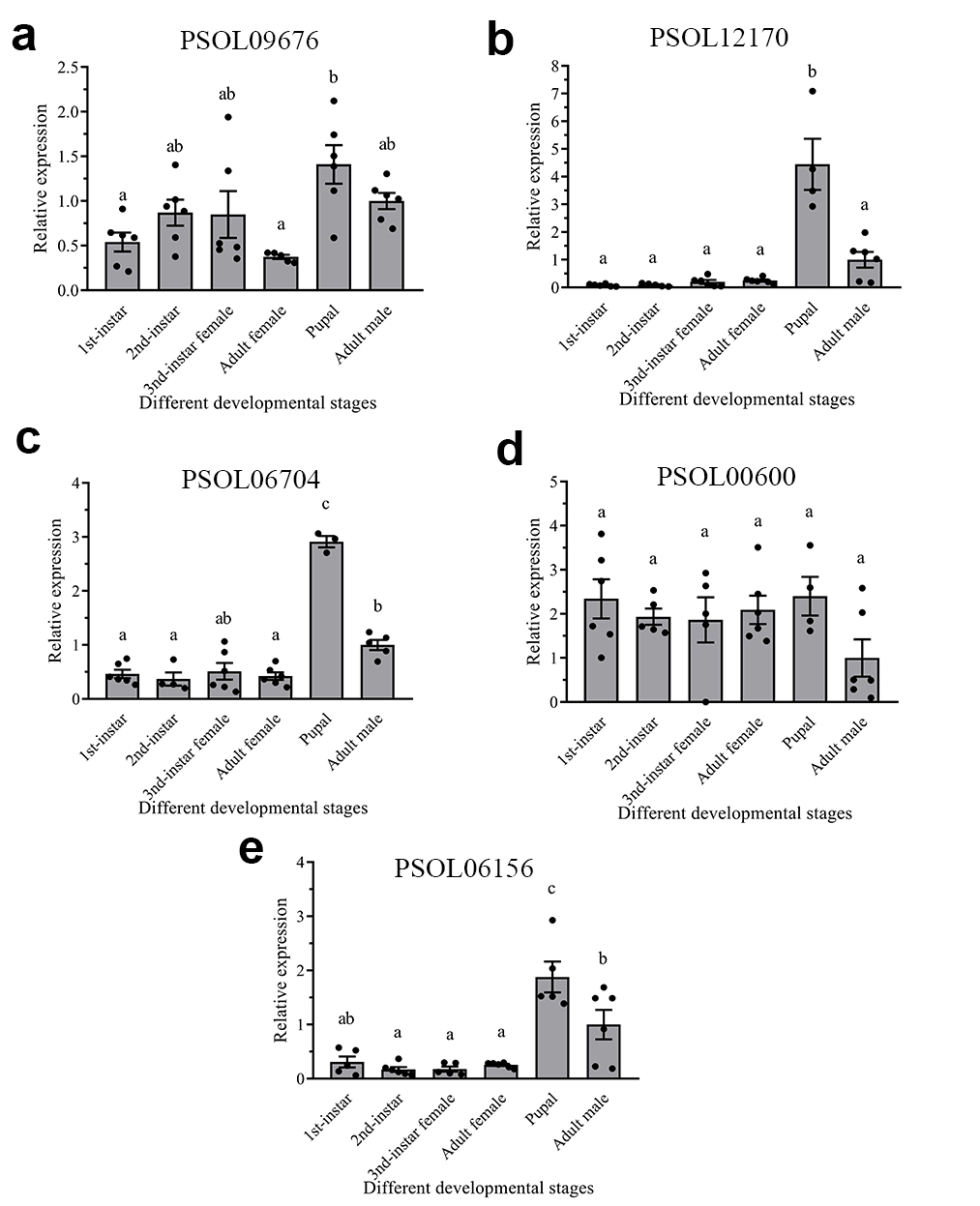


Supplementary Figure 3 Developmental expression profiles of five DEGs in cotton mealybugs. The gene ids are shown on the top of each histogram. Two *FAS* genes (**a**, **b**), one *ELO* gene (**c**) and one *FAR* gene (**e**) were dominantly expressed in pupal and male stages, whereas expression of the remaining *ELO* genes (**d**) showed no significant changes during development. The relative expression level was normalized and visualized as the means ± SEM with four biological replicates. Different lower-case letters indicate a significant difference (*P* < 0.05) based on ANOVA followed by Trukey’s multiple comparison test.


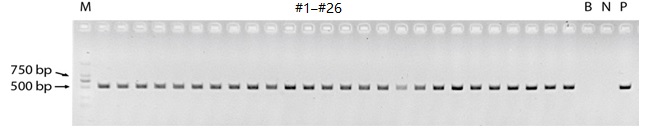


Supplementary Figure 4 Selection of positive tobacco transformants via PCR amplification of the *hpt* gene. The first lane is marker (M), followed by 26 transformants (#1–#26), blank control (B), negative control (N) and Positive control (P).


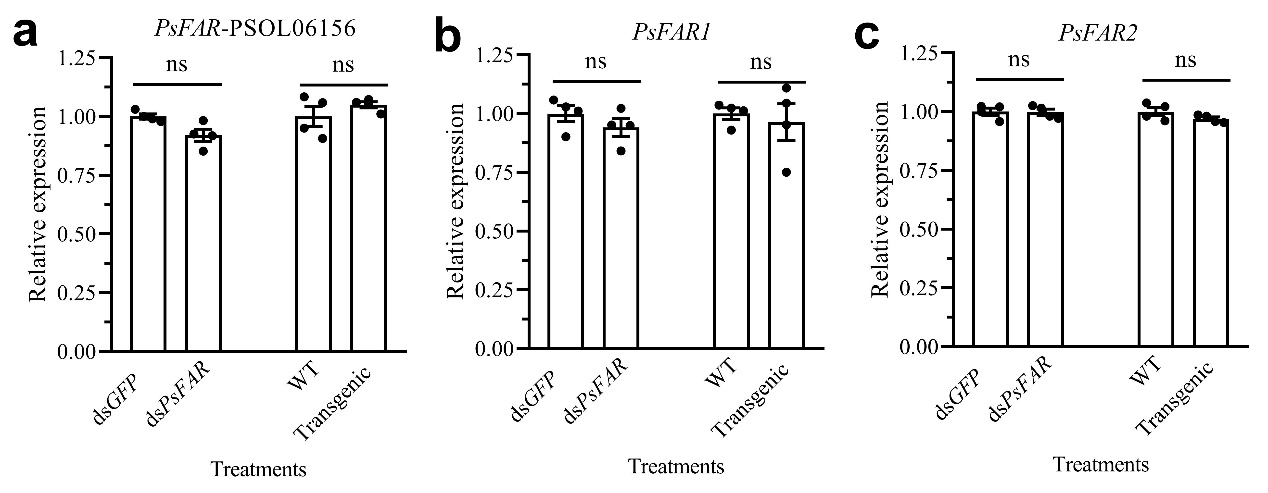


Supplementary Figure 5 Gene expression of *FAR* paralogs in cotton mealybug. **a** *PsFAR* identified in this study. **b** and **c** represent *PsFAR1* and *PsFAR2* identified by Li et al., (2016). The relative expression level was normalized and visualized as the means ± SEM with three biological replicates. WT: insects fed on wild type plants, Transgenic: insects fed on GM tobacco plants. ns denote no significant difference as determined by Student’s *t*-test with *P*= 0.05.


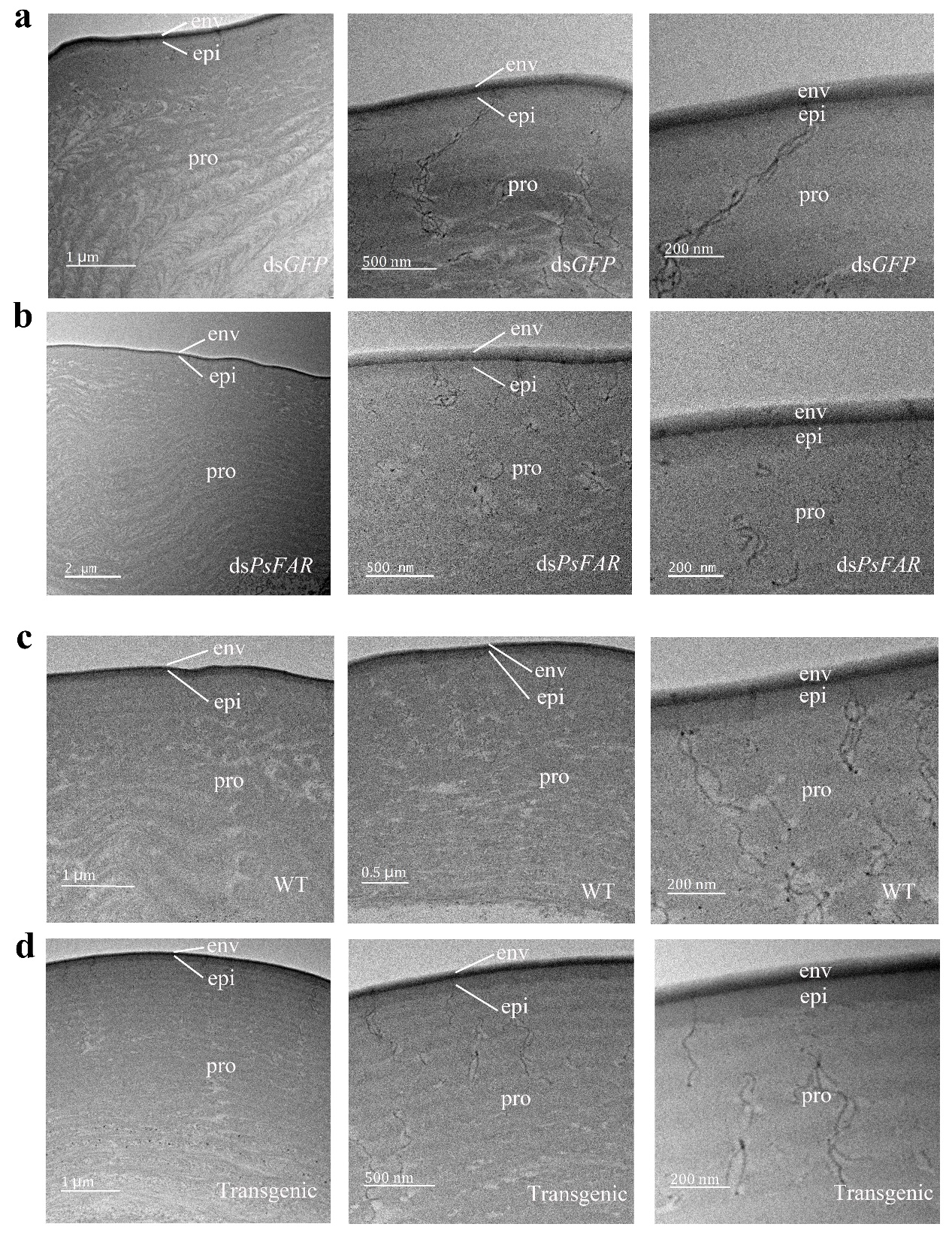


Supplementary Figure 6 TEM analysis on the integumentary envelope. env: envelope, epi: epicuticle, pro: procuticle. **a** ds*GFP* injection treated group. **b** ds*PsFAR* injection treated group. **c** WT tobacco rearing treated group. **d** Transgenic tobacco rearing treated group. WT: insects fed on wild type plants, Transgenic: insects fed on GM tobacco plants. 24 hours post emergence, live insects were collected for TEM.


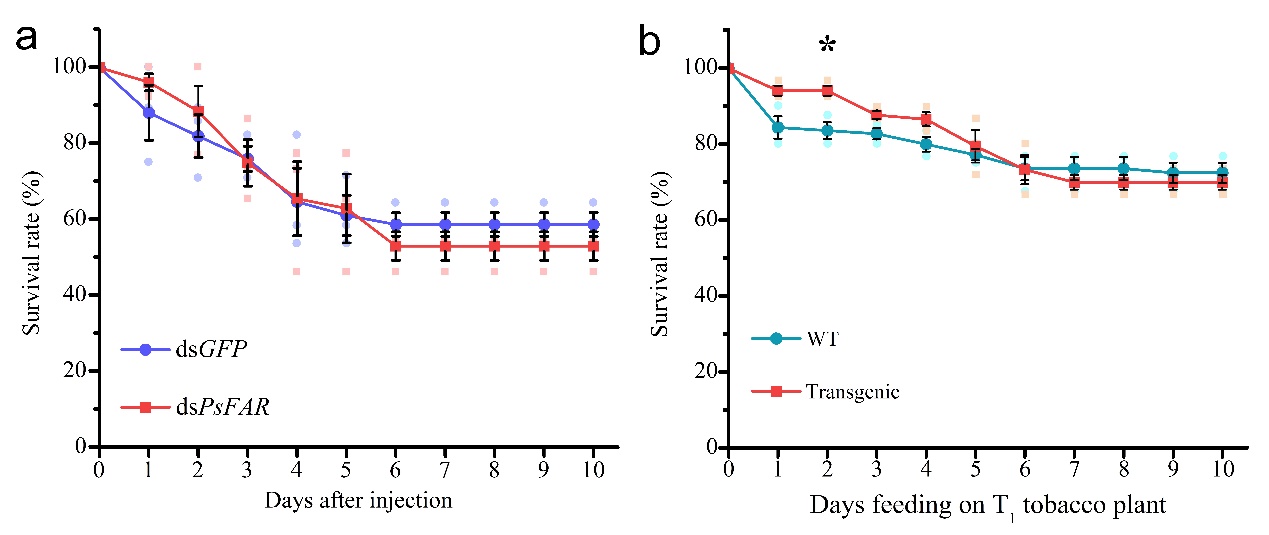


Supplementary Figure 7 Dynamic analysis of the survival rate of dsRNA injected (**a**) and tobacco fed (**b**) cotton mealybugs after spraying with water. WT: insects fed on wild type plants, Transgenic: insects fed on GM tobacco plants. ds*GFP* and WT were respectively set as negative controls, n = 30 insects. Means ± SEM were calculated with results from three biological replicates. Asterisks denote a significant difference as determined by Student’s *t*-test; ^*^ *P* < 0.05.


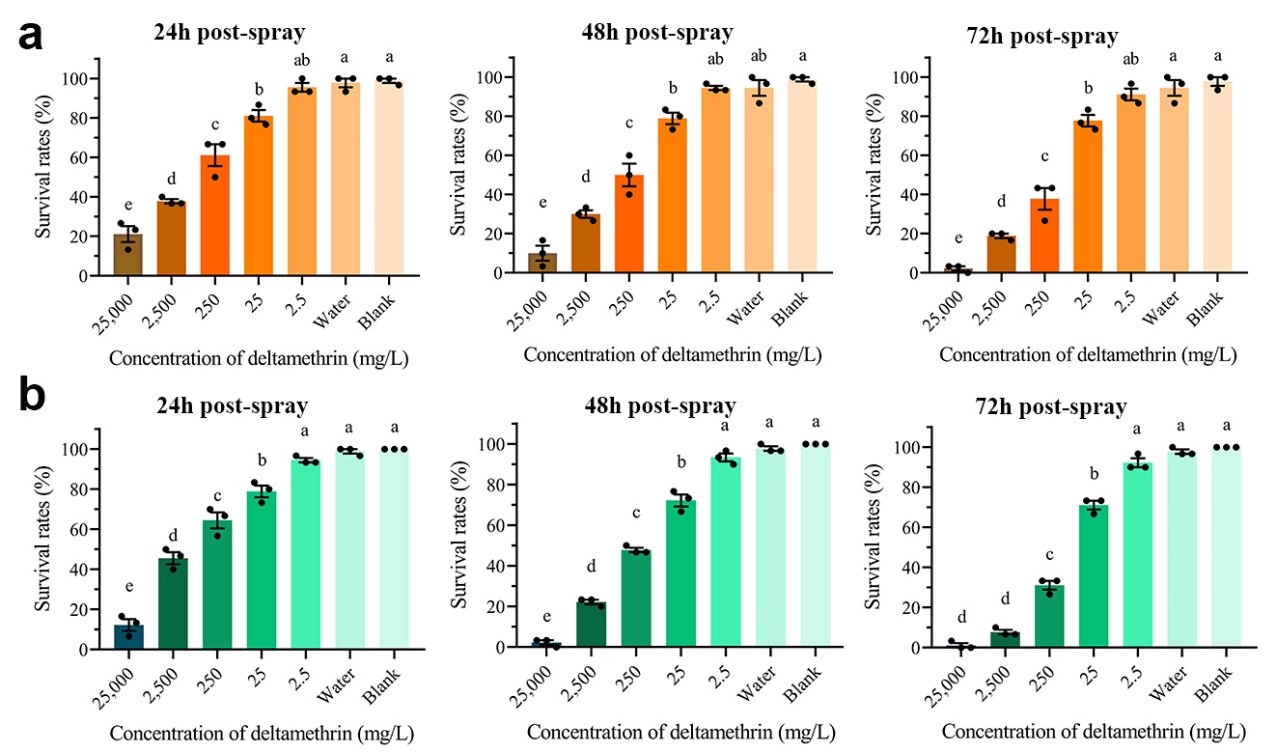


Supplementary Figure 8 Effects of different concentrations of deltamethirin on the survival rates of cotton mealybugs feeding on tomato (**a**) and tobacco (**b**). Histograms show the survival rates 24, 48, and 72 hours after pesticide treatment. n = 30 insects. Data are presented as means ± SEM from three biological replicates. Different letters indicate significant differences determined by one-way ANOVA followed by the Tukey test (*P* < 0.05).


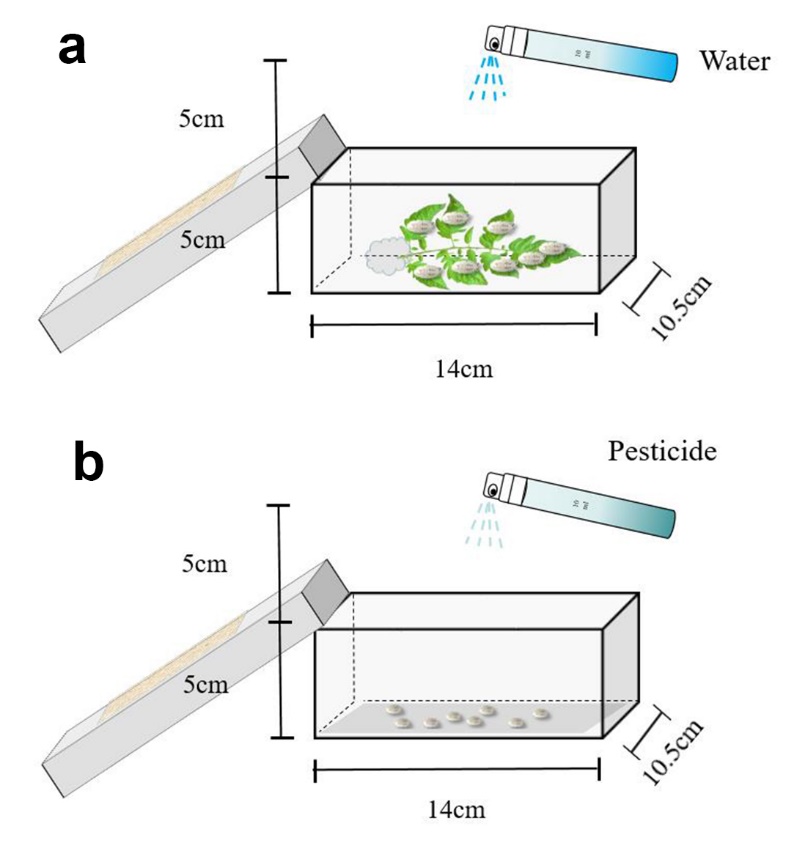


Supplementary Figure 9 Diagrammatic drawing of water (**a**) and insecticide (**b**) spray assays.

Supplementary Table 1 Information on the six RNA-seq datasets

| Samples | Raw Base (bp) | Clean Base (bp) | Clean Reads Rate (%) | Q30 (%) |
| --- | --- | --- | --- | --- |
| Integument_1 | 10,483,840,500 | 10,308,260,400 | 98.33 | 93.70 |
| Integument _2 | 9,545,312,400 | 9,372,926,400 | 98.19 | 94.44 |
| Integument _3 | 8,610,469,800 | 8,513,469,900 | 98.87 | 94.26 |
| Non-integumentary tissues_1 | 9,883,364,400 | 9,717,378,600 | 98.32 | 93.38 |
| Non-integumentary tissues_2 | 11,120,191,500 | 10,969,776,300 | 98.65 | 93.31 |
| Non-integumentary tissues_3 | 9,682,476,900 | 9,556,647,300 | 98.70 | 93.42 |

Supplementary Table 2 Count numbers of six DEGs participating in the hydrocarbon synthesis pathway

| Gene name | Gene ids | Mean counts | log_2_FC | *P*-value | *P*-adj | Integument_1 | Integument_2 | Integument_3 | Non-integumentary tissues_1 | Non-integumentary tissues_2 | Non-integumentary tissues_3 |
| --- | --- | --- | --- | --- | --- | --- | --- | --- | --- | --- | --- |
| FAS | PSOL09676 | 1137.74 | -2.1936 | 0.0004038 | 0.0094272 | 2028.00 | 2294.43 | 1280.03 | 159.46 | 740.764 | 323.768 |
| FAS | PSOL12170 | 38.32 | -1.6558 | 3.88E-05 | 0.0013132 | 65.52 | 50.33 | 58.09 | 23.03 | 14.62 | 18.34 |
| ELO | PSOL06704 | 2101.87 | -2.1631 | 4.50E-05 | 0.0015106 | 2536.55 | 5899.66 | 1873.97 | 708.69 | 988.57 | 603.80 |
| ELO | PSOL00600 | 5170.45 | -2.1550 | 7.98E-05 | 0.0024369 | 5914.47 | 12128.58 | 7291.57 | 1986.99 | 2834.17 | 866.91 |
| FAR | PSOL02039 | 4253.86 | -3.4700 | 4.82E-06 | 0.0002184 | 2850.63 | 16818.11 | 3741.94 | 829.17 | 875.63 | 407.71 |
| FAR | PSOL06156 | 201.65 | -3.9203 | 1.35E-07 | 9.18E-06 | 171.60 | 805.36 | 158.25 | 23.03 | 31.23 | 20.46 |

Supplementary Table 3 Blast results of six DEGs that mapped with the integumentary proteomics data

| Gene name | Gene ids | Transcript ids | Protein ids | e-value |
| --- | --- | --- | --- | --- |
| FAS | PSOL09676 | MSTRG.1735.1 | CUFF.3563.1-m.6410 | 0 |
| FAS | PSOL12170 | MSTRG.9664.1 | CUFF.13358.1-m.23974 | 3E-170 |
|  |  | MSTRG.9664.2 | CUFF.13363.2-m.24002 | 0 |
| ELO | PSOL06704 | MSTRG.874.1 | CUFF.14346.1-m.25819 | 0 |
|  |  | MSTRG.874.2 | CUFF.14346.1-m.25819 | 2E-92 |
|  |  | MSTRG.874.3 | CUFF.14346.1-m.25819 | 0 |
| ELO | PSOL00600 | MSTRG.7329.1 | CUFF.10396.2-m.18627 | 0 |
|  |  | MSTRG.7329.2 | CUFF.10396.2-m.18627 | 0 |
| FAR | PSOL02039 | MSTRG.2191.1 | CUFF.2437.2-m.4356 | 0 |
|  |  | MSTRG.2191.2 | CUFF.2438.1-m.4524 | 0 |
|  |  | MSTRG.2191.3 | CUFF.2437.2-m.4356 | 0 |
|  |  | MSTRG.2191.4 | CUFF.2466.1-m.4531 | 0 |
| FAR | PSOL06156 | MSTRG.2729.1 | CUFF.13241.2-m.23755 | 9E-175 |
|  |  | MSTRG.2729.2 | CUFF.13241.3-m.23756 | 7E-175 |

Supplementary Table 4 Effect of *PsFAR* knockdown on CHC content of cotton mealybugs

| Retention Time | Compound | ds*GFP* | ds*PsFAR* | WT | Transgenic |
| --- | --- | --- | --- | --- | --- |
|  | Total CHCs | 11.49 ± 2.09 | 4.63 ± 0.70^*^ | 10.94 ± 1.15 | 4.64 ± 0.76^**^ |
| 5.87 | *n*-C_9_ | 0.91 ± 0.28 | 0.68 ± 0.26 | 0.80 ± 0.18 | 0.80 ± 0.34 |
| 8.07 | *n*-C_10_ | - | - | 0.91 ± 0.39 | 0.75 ± 0.74 |
| 9.94 | *n*-C_11_ | 0.23 ± 0.06 | 0.07 ± 0.01 | 0.13 ± 0.04 | 0.63 ± 0.58 |
| 13.06 | *n*-C_13_ | 2.06 ± 0.60 | 0.91 ± 0.33 | 2.14 ± 0.36 | 0.88 ± 0.55 |
| 14.42 | *n*-C_14_ | 0.38 ± 0.22 | 0.27 ± 0.23 | 0.05 ± 0.02 | 0.11 ± 0.04 |
| 15.69 | *n*-C_15_ | 1.26 ± 0.44 | 0.53 ± 0.17 | 0.50 ± 0.26 | 0.32 ± 0.09 |
| 16.89 | *n*-C_16_ | 1.90 ± 0.49 | 0.40 ± 0.15^**^ | 0.33 ± 0.20 | 0.61 ± 0.26 |
| 18.03 | *n*-C_17_ | 0.79 ± 0.39 | 0.10 ± 0.03 | 0.04 ± 0.01 | 0.11 ± 0.03 |
| 19.11 | *n*-C_18_ | 0.20 ± 0.06 | 0.02 ± 0.01^*^ | 0.03 ± 0.01 | 0.10 ± 0.04 |
| 20.14 | *n*-C_19_ | 0.07 ± 0.03 | 0.05 ± 0.01 | 0.37 ± 0.11 | 0.06 ± 0.03^*^ |
| 21.12 | *n*-C_20_ | 1.57 ± 0.92 | 1.46 ± 0.69 | 2.96 ± 0.40 | 1.40 ± 0.64 |
| 22.96 | *n*-C_22_ | 1.07 ± 0.59 | 0.80 ± 0.44 | 1.91 ± 0.60 | 1.20 ± 0.67 |
| 23.82 | *n*-C_23_ | - | - | 0.14 ± 0.03 | 0.09 ± 0.06 |
| 24.65 | *n*-C_24_ | 0.19 ± 0.02 | 0.07 ± 0.04 | 0.18 ± 0.09 | 0.13 ± 0.07 |
| 25.45 | *n*-C_25_ | - | - | 0.12 ± 0.07 | 0.04 ± 0.03 |
| 26.22 | *n*-C_26_ | 0.12 ± 0.06 | 0.01 ± 0.00 | 0.01 ± 0.00 | 0.00 ± 0.00 |
| 26.96 | *n*-C_27_ | - | - | 0.07 ± 0.02 | 0.06 ± 0.04 |
| 27.68 | *n*-C_28_ | 0.25 ± 0.06 | 0.00 ± 0.00^**^ | 0.02 ± 0.01 | 0.02 ± 0.01 |
| 28.38 | *n*-C_29_ | - | - | 0.12 ± 0.05 | 0.01 ± 0.00^*^ |
| 29.05 | *n*-C_30_ | 0.12 ± 0.04 | 0.00 ± 0.00^*^ | 0.23 ± 0.10 | 0.01 ± 0.00 |
| 29.70 | *n*-C_31_ | 0.01 ± 0.01 | 0.01 ± 0.00 | 0.00 ± 0.00 | 0.00 ± 0.00 |
| 31.06 | *n*-C_33_ | 0.09 ± 0.05 | 0.00 ± 0.00 | 0.00 ± 0.00 | 0.02 ± 0.01 |
| 32.80 | *n*-C_35_ | 0.01 ± 0.01 | 0.01 ± 0.00 | 0.00 ± 0.00 | 0.06 ± 0.04 |
| 35.17 | *n*-C_37_ | 0.02 ± 0.01 | 0.01 ± 0.00 | 0.02 ± 0.01 | 0.01 ± 0.01 |

Results were calculated from six biological replicates (nanogram per milligram of fresh body mass ± SEM). Asterisks ^*^ and ^**^ respectively indicate significant differences at *P* < 0.05 and *P* < 0.01 using Student’s *t*-test, between ds*GFP* and ds*PsFAR* treatments, or between WT and Transgenic groups. WT: insects fed on wild type plants, Transgenic: insects fed on GM tobacco plants.

Supplementary Table 5 Effect of *PsFAR* knockdown on the survival days and survival rates of cotton mealybugs

| Assays | Treatments | ds*GFP* | ds*PsFAR* | WT | Transgenic |
| --- | --- | --- | --- | --- | --- |
| Desiccation assay | 75 % humidity | 8.16 ± 0.36 | 7.42 ± 0.45 | 8.93 ± 0.45 | 8.37 ± 0.35 |
|  | <10 % humidity | 3.66 ± 0.15 | 3.14 ± 0.15^*^ | 4.64 ± 0.19 | 3.48 ± 0.14^**^ |
| Contact killing assay | No deltamethrin spray | 94.48 ± 1.04 | 88.80 ± 1.06^*^ | 95.56 ± 1.11 | 86.67 ± 1.93^*^ |
|  | Sprayed with 25 mg/L deltamethrin | 80.63 ± 1.41 | 64.07 ± 1.15^**^ | 72.22 ± 2.94 | 50.00 ± 1.92^**^ |

Days ± SEM and survival rates ± SEM were respectively showed for the results of desiccation and contact killing assays. All results were calculated from three biological replicates. *P*-values are indicated using Student’s *t*-test. The asterisks ^*^ and ^**^ indicate significant differences at *P* < 0.05 and *P* < 0.01, respectively, between ds*GFP* and ds*PsFAR* treatments, or between WT and transgenic groups. WT: insects fed on wild type plants, Transgenic: insects fed on GM tobacco plants.

Supplementary Table 6 Primers used in the study

| Purpose | Primer name | Primer sequence |
| --- | --- | --- |
| RT-qPCR | PsFAS-PSOL09676-F | CGCAGGTTACAATCCGCAAC |
|  | PsFAS-PSOL09676-R | GCGGCATATTCGTTTGCTGT |
|  | PsFAS-PSOL12170-F | TGGCCTACACAGCGATTCAA |
|  | PsFAS-PSOL12170-R | AGCGTCGAAAATAGCGGTGA |
|  | PsELO-PSOL06704-F | AGGTGCTGTAGTTGGTTGGA |
|  | PsELO-PSOL06704-R | TGCATTTCAGGACCCAATGC |
|  | PsELO-PSOL00600-F | CGTGGTTGTTCTACGAGGCT |
|  | PsELO-PSOL00600-R | GGCAGCCGGTGAATAAGAGT |
|  | PsFAR-PSOL02039-F | TGGTGGTACTGGATTCATGGG |
|  | PsFAR-PSOL02039-R | CGTCCGGCGATTTTCCTTTC |
|  | PsFAR-PSOL06156-F | AAAAGGACAATCATCGCAAGAGA |
|  | PsFAR-PSOL06156-R | GGCTGACCAAGATCACCACTC |
|  | PsActin-F | TCGTACCACCGGTATCGTATTA |
|  | PsActin-R | TTAAGTCACGACCAGCCAAG |
| RACE | PsFAR-5’end | AAGTATGCGTAATGCCACACGCAC |
|  | PsFAR-3’end | AGTTCTAGTGCGTGTGGCATTACGCA |
| dsRNA synthesis^*^ | dsPsFAR-F | **TAATACGACTCACTATAGGG**GTGTGATTGTGGGAGTCGGT |
|  | dsPsFAR-R | **TAATACGACTCACTATAGGG**TCTCCCGCACATCGTTCATC |
| pCAMBIA1301-ds*PsFAR* construction^*^ | BamHI-SalI-dsPsFAR-F | CGCggatccgtcgacACGGCTTATTCCTTCTGCC |
|  | SacI-ApaI-dsPsFAR-R | GGgagctcgggcccGGTTGCTTTAGCCGCACAA |
| Selection of ds*PsFAR* GM tobacco plants | hpt557-F | ACACTACATGGCGTGATTTCAT |
|  | hpt557-R | TCCACTATCGGCGAGTACTTCT |
|  | RbcL-F | ATGTCACCACAAACAGAGACTAAAGC |
|  | RbcL-R | GAAACGGTCTCTCCAACGCAT |
| ds*PsFAR* expression in GM tobacco plants | gm-PsFAR-F | TTTGCCTGTTGGCGTGTTTC |
|  | gm-PsFAR-R | CAATCGGTCCATACACATTG |
|  | EF-1A-F | AGCTTCACCTCCCAGGTCATC |
|  | EF-1A-R | AGAACGCCTGTCAATCTTGG |

^*^ The T7 RNA polymerase promoter is in bold. Minuscule representation of restriction enzyme sequences.
