# Supplementary material for: Fatty acyl-CoA reductase influences wax biosynthesis in the cotton mealybug, Phenacoccus solenopsis Tinsley
Source: Commun Biol. 2022 Oct 19;5:1108. doi: 10.1038/s42003-022-03956-y (PMC9582030; doi:10.1038/s42003-022-03956-y)
Supplement: Supplementary file 3 — Description of Additional Supplementary Files [file 42003_2022_3956_MOESM3_ESM.pdf]

Description of Additional Supplementary Files

File name: Supplementary Data 1
Description: Identified chemical compounds (top 20) in the wax of cotton mealybugs under different treatments.

File name: Supplementary Data 2
Description: Transcriptomic data of Integument and non-integument.

File name: Supplementary Data 3
Description: Transcriptomic data of Integument and non-integument.

File name: Supplementary Data 4
Description: The source data underlying graphs, plots, and charts.
