# Supplementary material for: Fatty acyl-CoA reductase influences wax biosynthesis in the cotton mealybug, Phenacoccus solenopsis Tinsley
Source: Commun Biol. 2022 Oct 19;5:1108. doi: 10.1038/s42003-022-03956-y (PMC9582030; doi:10.1038/s42003-022-03956-y)
Supplement: Supplementary file 8 — Reporting Summary [file 42003_2022_3956_MOESM8_ESM.pdf]

nature research

Corresponding author(s): Fei Li

Last updated by author(s): Aug 16, 2022

Reporting Summary

Nature Research wishes to improve the reproducibility of the work that we publish. This form provides structure for consistency and transparency in reporting. For further information on Nature Research policies, see our Editorial Policies and the Editorial Policy Checklist.

Statistics

For all statistical analyses, confirm that the following items are present in the figure legend, table legend, main text, or Methods section.

n/a	Confirmed	
☐	☒	The exact sample size (*n*) for each experimental group/condition, given as a discrete number and unit of measurement
☐	☒	A statement on whether measurements were taken from distinct samples or whether the same sample was measured repeatedly
☐	☒	The statistical test(s) used AND whether they are one- or two-sided<br>*Only common tests should be described solely by name; describe more complex techniques in the Methods section.*
☒	☐	A description of all covariates tested
☐	☒	A description of any assumptions or corrections, such as tests of normality and adjustment for multiple comparisons
☐	☒	A full description of the statistical parameters including central tendency (e.g. means) or other basic estimates (e.g. regression coefficient) AND variation (e.g. standard deviation) or associated estimates of uncertainty (e.g. confidence intervals)
☐	☒	For null hypothesis testing, the test statistic (e.g. *F*, *t*, *r*) with confidence intervals, effect sizes, degrees of freedom and *P* value noted<br>*Give P values as exact values whenever suitable.*
☒	☐	For Bayesian analysis, information on the choice of priors and Markov chain Monte Carlo settings
☒	☐	For hierarchical and complex designs, identification of the appropriate level for tests and full reporting of outcomes
☒	☐	Estimates of effect sizes (e.g. Cohen's *d*, Pearson's *r*), indicating how they were calculated

Our web collection on statistics for biologists contains articles on many of the points above.

Software and code

Policy information about availability of computer code

Data collection	SPSS 19, GraphPad Prism 8.0, and Excel
Data analysis	SPSS 19, GraphPad Prism 8.0, and Excel

For manuscripts utilizing custom algorithms or software that are central to the research but not yet described in published literature, software must be made available to editors and reviewers. We strongly encourage code deposition in a community repository (e.g. GitHub). See the Nature Research guidelines for submitting code & software for further information.

Data

Policy information about availability of data

All manuscripts must include a data availability statement. This statement should provide the following information, where applicable:

- Accession codes, unique identifiers, or web links for publicly available datasets
- A list of figures that have associated raw data
- A description of any restrictions on data availability

The RNA-seq data of integumentary and non-integumentary tissues have been deposited in GenBank with the accession numbers PRJNA798788. The proteome data of the integument have been deposited in PeptideAtlas under the accession number PASS01729.

Field-specific reporting

Please select the one below that is the best fit for your research. If you are not sure, read the appropriate sections before making your selection.

☒ Life sciences ☐ Behavioural & social sciences ☐ Ecological, evolutionary & environmental sciences

For a reference copy of the document with all sections, see nature.com/documents/nr-reporting-summary-flat.pdf

Life sciences study design

All studies must disclose on these points even when the disclosure is negative.

Sample size	For wax chemical composition analysis, sample size n=1000; RNA-seq, sample size n=150; proteome sequencing, sample size n=300; assessing RNAi, sample size n=7 for microinjection and n=10 for tobacco feeding; extraction of CHCs, sample size n=50; desiccation assay, sample size n=30–34; SEM analysis for wax reduction, water proofing assay and insecticide assay, sample size n=30; TEM for integument, sample size n=5
Data exclusions	All the data does not meet the acceptance criteria and all the outliers are excluded
Replication	For wax chemical composition analysis and proteome analysis, replication n=1; expression profiles of PsFAR, replication n=4; others, replication n=3
Randomization	Animal study are randomized
Blinding	Animal study are double-blinded

Reporting for specific materials, systems and methods

We require information from authors about some types of materials, experimental systems and methods used in many studies. Here, indicate whether each material, system or method listed is relevant to your study. If you are not sure if a list item applies to your research, read the appropriate section before selecting a response.

Materials & experimental systems

n/a	Involved in the study	
☒	☐	Antibodies
☒	☐	Eukaryotic cell lines
☒	☐	Palaeontology and archaeology
☐	☒	Animals and other organisms
☒	☐	Human research participants
☒	☐	Clinical data
☒	☐	Dual use research of concern

Methods

n/a	Involved in the study	
☒	☐	ChIP-seq
☒	☐	Flow cytometry
☒	☐	MRI-based neuroimaging

Animals and other organisms

Policy information about studies involving animals; ARRIVE guidelines recommended for reporting animal research

Laboratory animals	Phenacoccus solenopsis Tinsley were originally collected from Rose of Sharon, Hibiscus syriacus L. (Malvales: Malvaceae) in Jinhua, China, in June 2016. They were maintained on fresh tomato plants (cv. Hezuo-903, Shanghai Changzhong Seeds Industry Co., Ltd, China) in a climatically controlled chamber maintained at 27 ± 1 °C, 75 % relative humidity (RH), and a photoperiod of 14:10 (L:D)
Wild animals	not applicable
Field-collected samples	not applicable
Ethics oversight	not applicable

Note that full information on the approval of the study protocol must also be provided in the manuscript.
